# Unraveling the relationship between inflammation and cluster headache

**DOI:** 10.3389/fneur.2025.1548522

**Published:** 2025-04-03

**Authors:** Yu-Wen Wang, Xu-Hong Yang, Xin-Hui Zheng, Gao-Shui Zhou, Xiao-Xia Zhao, Yi-Lan Zhao, Shu-Hong Wu

**Affiliations:** ^1^Chengdu University of Traditional Chinese Medicine, Chengdu, Sichuan, China; ^2^Chongqing Beibei District Hospital of Traditional Chinese Medicine, Chongqing, China

**Keywords:** cluster headache, neurogenic inflammation, trigeminal-vascular system, neuropeptides, inflammatory mediators

## Abstract

Cluster headache (CH) is often referred to as the ‘suicide headache.’ Existing research suggests that the activation of the trigeminal-vascular system, increased sensitivity of nerve fibers, and the release and interaction of various neuropeptides and inflammatory mediators may contribute to neurogenic inflammation, which serves as a crucial pathophysiological basis for the development of CH. Additionally, some neuropeptides can modulate neuronal activity related to pain transmission and may increase pain perception by sensitizing central nerves. This review discusses the neuropeptides and inflammatory mediators associated with CH neuroinflammation, focusing on calcitonin gene-related peptide (CGRP), inflammatory cytokines and related signaling pathways, nitric oxide (NO), pituitary adenylate cyclase-activating peptide 38 (PACAP-38), and vasoactive intestinal peptide (VIP), incorporating both preclinical and clinical evidence to provide new insights into potential therapeutic targets for CH.

## 1 Introduction

Cluster Headache (CH) is a primary trigeminal autonomic headache (TAC) that can be divided into episodic cluster headache (ECH) and chronic cluster headache (CCH). ECH is characterized by recurrent headache bouts lasting from 7 days to 1 year, with pain-free intervals of at least 3 months between episodes. In contrast, CCH involves headaches occurring for over a year without a complete pain-free period, or with pain-free intervals shorter than 3 months. It is clinically characterized by severe unilateral pain in the orbital, supraorbital, and temporal regions. This pain is always accompanied by cranial autonomic symptoms and typically lasts between 15 and 180 min (1). CH imposes a significant burden on both individual lives and the national economy (2, 3). During the attack phase, patients with CH experience heightened levels of depression and hopelessness (4), with a notable increase in suicidal tendencies (5). Furthermore, the fear of CH attacks has emerged as an independent factor contributing to pain-related disability (6). In a recent large-scale case collection (7), only 11.89% of patients received an accurate diagnosis of CH during their initial visit, and one-third of the patients experienced a diagnostic delay of over 10 years. More than half of those with chronic CH met the criteria for refractoriness, and approximately 60.2% remained highly symptomatic despite treatment (8). The lifetime prevalence of the disease is estimated at 1.24 per 1,000 individuals (9), and the overall prognosis remains poor.

Neuroinflammation is mediated by proinflammatory cytokines, chemokines, second messengers, and reactive oxygen species (10), primarily involving the activation responses of microglia, astrocytes, and immune cells (11). A recent study analyzed inflammatory markers in the cerebrospinal fluid of CH patients and found that factor superfamily 10 (TNFSF10) and TNFSF12, along with several chemokines, were elevated to varying degrees, with minor differences observed between the active and remission phases (12). The elevated levels of inflammatory markers and chemokines in the cerebrospinal fluid suggest the possibility of persistent neuroinflammation in the central nervous system (CNS) of CH patients, rather than it being confined solely to the active phase. Neurogenic inflammation, defined as ‘acute aseptic inflammation,’ is primarily triggered by neuropeptide mediators released from injured peripheral sensory fibers (13), which is accompanied by the local activation of mast cells that promote the release of proinflammatory cytokines and other inflammatory mediators (14). The involvement of neurogenic inflammation in the pathogenesis of migraine has been substantiated by extensive preclinical experiments and clinical studies (14, 15). However, the relationship between CH and neurogenic inflammation can only be inferred from the alterations in related inflammatory mediators and neuropeptides observed in clinical studies involving CH patients. In this paper, we present various neuropeptides and inflammatory mediators linked to neuroinflammation in CH and infer their roles in the pathogenesis of inflammation related to CH, aiming to provide new insights that lead to potentially effective therapeutic targets for future clinical applications.

## 2 Trigeminal vascular system and neurogenic inflammation

The trigeminal vascular system comprises the trigeminal nerve (CNV), associated nuclei, and cranial vessels (16), which are primarily influenced by three types of nerve fibers (17). The first type is trigeminal sensory fibers, which release calcitonin gene-related peptide (CGRP), substance P (SP), nitric oxide (NO), and pituitary adenylate cyclase-activating peptide (PACAP). The second type consists of parasympathetic fibers that release vasoactive intestinal peptide (VIP), PACAP, neuropeptide Y (NPY), acetylcholine, and NO. Lastly, sympathetic fibers release norepinephrine, adenosine triphosphate (ATP), and NPY. Specific neuropeptides are produced by the neurons of the trigeminal nerve and specifically innervate the intracranial vascular system. Upon stimulation of the trigeminal nerve, these neuropeptides are released, subsequently activating the associated nerve fibers. These neuropeptides do not encompass all neuropeptides of the parasympathetic, sympathetic, and sensory systems, but rather represent specific types that are closely associated with the function of the trigeminal nerve. This activation triggers a series of potential cascade responses that lead to the activation and sensitization of the trigeminal vascular system, which is particularly involved in the development of primary headaches. Preclinical studies have confirmed that trigeminal autonomic hyperalgesia is driven by brainstem activation, with parasympathetic pathways playing a significant role (18). Additionally, face and neck pain have been shown to be highly predictive of prodromal symptoms in CH (19). The pathophysiological basis of CH is primarily associated with the activation of the trigeminal vascular pathway, trigeminal autonomic reflexes, and hypothalamic modulation (20). Although the exact pathogenesis remains unclear, it is generally acknowledged that the trigeminal vascular system serves as a common pathway in headache development and is a crucial pathophysiological basis for CH.

The cell bodies of trigeminal cells are located in the trigeminal ganglion, with its peripheral fibers primarily found mainly in the first (ophthalmic) branch of the trigeminal nerve. These fibers form synaptic connections with the intracranial blood vessels and dura mater, which are associated with the provocation of pain (21, 22). Activation of the trigeminal vascular system triggers trigeminal autonomic reflexes, exhibiting autonomic activity that is closely related to pain in CH. Additionally, a variety of neuropeptides are involved in the activation process. Recent studies have indicated that COVID-19 vaccination may trigger CH attacks, particularly in individuals with a pre-existing history of CH or those with certain predispositions (23, 24). One study suggests that the COVID-19 vaccination may activate the trigeminocervical complex and induce an inflammatory response (25), leading to the onset of headache episodes. However, the precise mechanisms remain unclear, and it is hypothesized that factors such as immune responses, neuroinflammation, or disruptions to neurovascular function might contribute to these attacks. Further research is required to comprehensively understand the underlying mechanisms. Genetic locus testing related to CH has also suggested a potential role for inflammatory responses in trigeminal activation (26). Furthermore, inflammation may exacerbate headache symptoms by promoting glial cell activation and immune cell infiltration.

Neuroinflammation serves as a protective process; however, its persistence activates glial cells in the brain, leading to the release of proinflammatory cytokines, chemokines, and neuropeptides. This release initiates an inflammatory cascade response, which contributes to widespread chronic pain through central sensitization (27, 28). Among the known pathogenetic mechanisms associated with primary headaches, stimulation of trigeminal and parasympathetic activation triggers the release of various neuropeptides, including CGRP, SP, VIP, NO, and PACAP (29). Consequently, this results in neurogenic inflammatory responses characterized by vasodilation, extravasation of plasma proteins, and degranulation of mast cells within surrounding target tissues (15). High-flow oxygen therapy is recommended as a first-line treatment in the acute phase of CH according to international guidelines (1). Preclinical studies have confirmed that oxygenation inhibits plasma protein extravasation in the rat dura mater (30) and alleviates neurogenic inflammation. However, additional evidence is required to substantiate the findings of current magnetic resonance imaging (MRI) studies regarding the role of neurogenic inflammation (31).

## 3 Neuropeptide mediators and their roles in CH

### 3.1 Calcitonin gene-related peptide

Calcitonin gene-related peptide (CGRP) is one of the most potent biologically active factors derived from the trigeminal ganglion, known to induce vasodilation and tissue edema by acting on receptors located on meningeal smooth muscle cells (32). CGRP also modulates the activity of injury-sensing neurons involved in pain transmission and plays a role in both peripheral and central nerve sensitization (33–35), thereby exacerbating pain perception. In the context of the inflammatory response, CGRP exhibits a bidirectional regulatory role (36–39). Furthermore, CGRP can activate glial cells, particularly astrocytes and microglia, which can intensify neuroinflammation through the release of additional inflammatory factors (40). These mechanisms may contribute to the development of neurogenic inflammation in CH. In 2019, the European Headache Federation published guidelines regarding the use of CGRP monoclonal antibodies for the prevention of migraine attacks (41). CGRP receptor antagonists are employed as both acute and prophylactic agents (42), however, research on the application of CGRP in CH continues to evolve.

CGRP levels are elevated in the external jugular vein during CH attacks, suggesting that the trigeminal nervous system is activated and CGRP is released into the extracranial circulation (43). Some investigators have reported elevated baseline levels of CGRP in all types of CH (44). However, recent findings indicate that plasma CGRP levels may be decreased during ictal CH compared to controls. This discrepancy may be attributed to variations in assay methods, as the mid-half-life of CGRP in plasma is only 7–10 min (45). The typical autonomic symptoms of a CH attack are linked to the activation of the first branch of the trigeminal nerve, which is responsible for tear secretion via the ophthalmic pathway. In a study, researchers found that measurements of CGRP levels in tears during a CH attack revealed concentrations approximately 159 times higher than those found in plasmam, furthermore, CGRP levels decreased following the administration of acute-phase medications such as triptans (46). In the pathogenesis of migraine, triptans can selectively act on the 5-HT1B/1D receptors located on the trigeminal ganglion, initiating relevant intracellular signaling pathways that inhibit the release of CGRP from trigeminal ganglion neurons (47). Since CGRP is a pro-inflammatory mediator with potent vasodilatory effects, reducing CGRP release limits vasodilation, alleviates neurogenic inflammation, and subsequently relieves pain. Additionally, triptans can activate the relevant 5-HT receptors on intracranial blood vessels, acting on the smooth muscle and endothelial cells to reverse vasodilation and mediate vasoconstriction, thereby achieving the desired effect of migraine symptom relief (48). Specific modulation of the trigeminal ganglion has been shown to significantly inhibit central sensitization when sumatriptan is administered systemically (49). It is inferred that similar mechanisms may be present in CH. Notably, in ECH patients, CGRP levels during the attack phase are higher than in the remission phase, however, no significant difference in CGRP levels was observed between the attack phase and CCH patients (45). Despite discrepancies in some study results, the majority of studies indicate that plasma CGRP levels vary across different phases of CH, suggesting CGRP may participate in the neuroinflammatory process through cyclic variations in different episodic states of CH. CGRP may act as a key mediator in the neuroinflammatory process of CH, interacting with multiple inflammatory mediators. Furthermore, each mediator, including CGRP, might independently regulate neuroinflammation through distinct pathways.

Some researchers have found that intravenous infusion of CGRP triggers headache attacks during the active phase of ECH and CCH, but does not trigger attacks of remission ECH (49). This suggests that anti-CGRP drugs may be effective in treating CH. There is evidence suggesting that CGRP-targeted therapy, particularly monoclonal antibodies that block CGRP or its receptor, holds significant potential as an effective treatment for CH (50). It is important to note that the CGRP monoclonal antibody Galcanezumab improved the symptoms of patients with ECH, but its effectiveness was limited in patients with CCH (51–53). Specifically, Galcanezumab significantly reduced the frequency of attacks in ECH patients (54) and decreased headache occurrence during the active phase (55). Although the safety of Galcanezumab has been verified through metrics such as the adverse event rate (56), the therapeutic response in CCH patients was less favorable (57). CGRP(R) antibodies have shown efficacy in at least some CCH patients (58), the overall efficacy remained limited. Fremanezumab, another monoclonal antibody, has shown negative results in both CH subtypes (59), while Eptinezumab, effective in migraine (60), has yet to be studied in CH. It is hypothesized that CGRP-targeting antibodies may offer some positive effects in ECH, particularly during the active phase. This may be partly attributed to the direct and central involvement of CGRP in the neuroinflammatory processes that occur during this phase. In contrast, CCH is characterized by long-term, persistent neuroinflammation and adaptive changes within the nervous system, which could attenuate the efficacy of CGRP-targeting antibodies in the chronic phase. The continuous stimulation from chronic pain results in prolonged hyperactivation of both the brain and trigeminal nervous system, a process that may be mediated not only by CGRP but also by other neuroinflammatory mediators and factors, thereby limiting the effectiveness of CGRP-based treatments. Notably, the significantly higher plasma CGRP levels observed in remission of ECH compared to CCH (44), led to the hypothesis that recurrent exacerbations of CCH drive progressive CGRP depletion, which diminishes responsiveness to CGRP-targeting antibodies and partially accounts for interindividual variability in therapeutic outcomes. Additionally, the limited sample size of clinical trials and individual patient differences may contribute to the variability in treatment outcomes. In conclusion, the current findings suggest that while Galcanezumab may show promise in treating CH, its overall efficacy and long-term safety require further validation through larger, prospective clinical trials with extended follow-up periods.

Recent studies on the repeated injection of botulinum toxin A (Onabotulinumtoxin A) into the pterygopalatine ganglion (SPG) (61) and the application of occipital nerve stimulation (ONS) therapy (62, 63)present new avenues for the treatment of refractory CH. One study reported a 50% failure rate in patients undergoing invasive ONS procedures, influenced by clinical symptoms characteristic of CH, including early onset, smoking habits, and fluctuations related to seasonal or circadian rhythms (64). For patients with refractory CH who do not respond adequately to the aforementioned prophylactic treatments, Galcanezumab has demonstrated some positive outcomes (58, 65).

In conclusion, CGRP-targeted therapy, primarily through monoclonal antibodies, has demonstrated some efficacy in the treatment of ECH; however, they fail to provide significant relief of clinical symptoms in patients with chronic refractory CH. While studies may be confounded by variables such as differing drug dosages and the involvement of concomitant prophylactic treatments, it can be hypothesized that CGRP is merely one critical component of the inflammatory cascade involved in the pathogenesis of CH, primarily associated with ECH attacks. In contrast, CCH is influenced by a multitude of factors, including opioid overuse, delayed diagnosis, and recurrent episodes, suggesting a closer association with alternative inflammatory mechanisms.

### 3.2 Pituitary adenylate cyclase-activating peptide 38/vasoactive intestinal peptide

Pituitary adenylate cyclase-activating peptide (PACAP), a sensory vasodilatory neuropeptide, not only exhibits vasodilatory effects but has also been validated in preclinical models as potentially correlating with central sensitization (66). Additionally, PACAP activates astrocytes, thereby inducing nociceptive transmission (67). Vasoactive intestinal peptide (VIP), which is structurally homologous to PACAP (68), is implicated in the neuroinflammation that occurs upon activation of the trigeminal nervous system (69). This activation leads to dural vasodilation (70)and manifests symptoms of autonomic activation, such as conjunctival congestion and tearing. PACAP-38 and VIP are parasympathetic peptides, thus they are involved in autonomic symptoms.

PACAP-38, one of the isoforms of PACAP, is predominantly expressed in neuronal tissues and is notably present in the trigeminal ganglion (TG) of both humans and rats (71). Alongside vasoactive intestinal peptide (VIP), it induces transient dilation of meningeal arteries and plays a specific role in the activation and sensitization of trigeminal vascular neurons (72). Preclinical studies indicate that sumatriptan, effective during CH episodes, partially mitigates the activating and sensitizing effects of PACAP-38 and reduces periorbital pain anomalies (73). During CH episodes, elevated plasma concentrations of PACAP-38 and VIP have been observed (43, 74), suggesting the release of these two neuropeptides. Intravenous infusion of PACAP-38 and VIP has been shown to trigger CH episodes in both ECH and CCH patients, though in fewer than 50% of cases. However, this effect does not occur during the remission phase (75). These findings imply that exogenous PACAP-38 and VIP may act as triggers during the active phase of CH, while their influence appears to be limited during remission, indicating that these neuropeptides may be more involved in the initiation of CH attacks rather than the maintenance of a persistent pathological state, though their effect appears less pronounced compared to CGRP. Further research has shown that plasma PACAP-38 concentrations are significantly higher during the attack phase in ECH patients, while they tend to decrease during periods of remission (74). Additionally, PACAP infusion induces a slight rise in plasma VIP levels during both active and remission phases in ECH patients, a phenomenon not observed in CCH patients (76). These dynamic changes may once more indicate that the two neuropeptides play distinct roles in regulating different CH subtypes or disease stages.

Notably, an experimental study demonstrates that VIP and PACAP can bind to the VPAC1 receptor to activate the cAMP/PKA pathway, suppressing NF-κB nuclear translocation and chemokine expression in microglia, thereby reducing peripheral immune cell recruitment, highlighting their potential anti-neuroinflammatory effects (77). However, current clinical studies have small sample sizes and high individual variability. Future work should integrate large-scale multi-omics analyses to clarify the molecular mechanisms underlying the distinct effects of PACAP/VIP in CCH versus ECH, with particular attention to their synergistic or antagonistic interactions with other key mediators like CGRP.

Some researchers have proposed that the role of PACAP in CH extends beyond merely inducing VIP release (17), as PACAP can be released during the activation of the trigeminal nervous system alongside parasympathetic nerves, whereas VIP primarily operates within the parasympathetic domain. Nonetheless, autonomic reflexes, such as conjunctival congestion triggered by the activation of the first branch of the trigeminal nerve, are closely associated with parasympathetic activation and the concurrent release of VIP. It is hypothesized that the activation of the trigeminal vascular pathway and trigeminal autonomic reflexes may contribute to the pathogenesis of CH through a vicious cycle (20). However, it remains unclear whether changes in VIP concentration could cause cross-reactivity when exogenous PACAP induces trigeminal and parasympathetic activation, where “cross-reactivity” refers to a series of unintended or additional responses triggered by changes in VIP concentrations during PACAP-induced trigeminal and parasympathetic activation. These responses may interfere with the accurate assessment of PACAP’s effects or produce other physiological impacts.

It has been demonstrated that neither plasma PACAP-38 nor VIP exhibited significant changes from baseline levels when CH episodes were induced by CGRP infusion (44).Additionally, plasma CGRP levels remained unchanged during PACAP-38/VIP-induced CH episodes (78), and plasma VIP concentrations similarly yielded negative results (76). While some animal studies suggest that PACAP-38 may induce CGRP release at certain sites, the correlation between PACAP and CGRP activation and release within the trigeminal vascular system appears to be weak (79). Both PACAP and CGRP are implicated in neurogenic inflammation, vasodilation, and pain sensation in migraine, albeit through distinct excitation and induction pathways (80). It remains unclear whether CGRP, PACAP, and VIP mediate CH pathogenesis via the same signaling pathway. Thus, targeting therapy by inhibiting PACAP-38 and VIP may represent a novel approach for CH patients who do not respond to anti-CGRP treatments. The PAC1 receptor monoclonal antibody, which has a high affinity for PACAP-38 (81), showed some possibility in preclinical migraine studies (82). Nevertheless, a phase II antibody clinical trial failed to demonstrate efficacy in migraine prevention (83), highlighting a critical gap between preclinical and clinical outcomes. While *in vitro* experiments suggest limited therapeutic responses when targeting a single PACAP receptor (84), evidence from animal models implies that dual modulation of VPAC1 and PAC1 receptors may enhance headache-related responses (73). Despite this potential, the combined targeting strategy has not yet been validated as a therapeutic approach for primary headaches. Further studies are urgently needed to resolve these discrepancies and explore whether dual-receptor antagonism could overcome the limitations observed in both preclinical and clinical settings. Given that trigeminal autonomic reflexes, originating from the trigeminal nerve, are modulated by PACAP and VIP-induced parasympathetic activation, they play a more prominent role in CH than in migraine. Future investigations of this class of targets in CH may reveal even greater therapeutic advantages.

## 4 Major inflammatory mediators in CH neuroinflammation

### 4.1 Inflammatory cytokines and related signaling pathways

#### 4.1.1 Interleukin IL-1 beta

Cytokines, as small soluble polypeptide proteins, play a crucial role in neuroinflammation. An imbalance between pro-inflammatory and anti-inflammatory cytokines contributes to this neuroinflammatory state. *In vitro* experiments have demonstrated that interleukin IL-1 beta (IL-1β) in brain tissue can be protective for neurons; however, an excessive inflammatory response induced by IL-1β may lead to neural damage (85). Methylprednisolone (MPD), a prophylactic drug for CH, exerts short-term protective effects by inhibiting IL-1β-induced activation of the trigeminal nerve in rat models (86). A study has found that serum levels of IL-1β are elevated in CH patients compared to controls during both the attack and remission phases and are particularly pronounced during the attack phase (87). The authors suggest that this elevation may represent an activation of the immune system. IL-1β may act as a positive feedback signal within the immune system, modulating the activity of other cytokines and immune cells involved in the inflammatory response to CH, and may also contribute to the modulation of pain through its interactions with neuropeptides. CGRP is known to be a neuropeptide involved in neuroinflammatory and pain responses. In cultured trigeminal neurons, IL-1β has been shown to induce the release of CGRP in trigeminal ganglia (88), while CGRP similarly promotes increased release of IL-1β (89), suggesting that these two molecules may be in a positive feedback loop. The interaction between IL-1β and CGRP synergistically enhances inflammation and has been found to be significantly relevant in migraine headaches (90, 91). However, the limited sample size in existing studies necessitates further research to substantiate the hypothesis that the IL-1β pathway plays a role in the neuroinflammatory response associated with CH.

A recent review highlights the therapeutic potential of IL-1β axis modulation in migraine, where IL-1 antagonists like Anakinra may suppress neuroinflammatory cascades to relieve the pain (92). Given that IL-1β’s mechanism in two diseases involves both the trigeminal ganglion and vascular path, and its elevation is observed in CH, IL-1 antagonists may hold similar therapeutic potential for CH. However, CH’s episodic nature and rapid progression may emphasize the need for time-sensitive therapeutic interventions in targeted research. Future studies should prioritize elucidating dynamic IL-1β signaling changes during active and remission phases, while investigating its synergistic interactions with other inflammatory mediators.

#### 4.1.2 S100B and NF-κB signaling pathway

S100 calcium-binding protein B (S100B) is a calcium-dependent protein primarily found in astrocytes and serves as a marker of glial activity. It acts as a pro-inflammatory protein in various neuroinflammatory diseases (93). Nuclear factor κB (NF-κB), a transcription factor involved in inflammation, immunity, and pain (94), plays an important role in the neuroinflammatory processes of nervous system diseases. A review study summarizes that physiological S100B levels show no significant effect on glial cell homeostasis; however, elevated levels promote a pro-inflammatory glial phenotype through NF-κB pathway activation, which enhances the secretion of inflammatory mediators such as IL-1β. Meanwhile, suppression of S100B expression reduces NF-κB activity and alleviates inflammatory responses (95). It suggests that S100B may regulate neuroinflammation through dynamic changes in concentration, with the involvement of the NF-κB pathway. A clinical study found that plasma levels of S100B are elevated in patients with ECH during the active phase compared to those with CCH. Furthermore, patients who experienced an attack following CGRP infusion had significantly higher baseline levels of S100B compared to those who did not (96). When glial cells are activated in response to painful stimuli, they release pro-inflammatory cytokines that amplify the inflammatory response. Therefore, elevated S100B levels reflect underlying neuroinflammatory processes, which may play a role in the pathophysiology of CH. Collectively, it can be inferred from these studies that S100B may serve to assess both CH attack activity and inflammatory intensity, as evidenced by its stage-dependent concentration changes. However, these findings require validation through further studies.

A study reported elevated levels of IL-1β and NF-κB in peripheral blood during CH attacks (97), consistent with previously observed inflammatory states. However, this study also found reduced levels of inflammasome complex components and S100B in patients exhibiting three or more autonomic neuropathies, such as conjunctival injection, rhinorrhea, eyelid edema, and myosis/ptosis. The activation of inflammasome complex components (e.g., NLRP3) typically induces the release of inflammatory mediators like IL-1β, directly initiating a pro-inflammatory signaling cascade. However, the observed changes in the levels of IL-1β and inflammasome complex components in the peripheral blood of patients with CH seem to be contradictory. Although self-regulation due to excessive inflammasome complexes activity cannot be excluded, these findings more likely suggest that the release of IL-1β is not dependent on inflammasome activation. Instead, alternative mechanisms such as NF-κB-mediated transcriptional activation may drive IL-1β involvement in CH pathogenesis. In addition, plasma S100B levels were elevated during the active phase of CH patients (96) but decreased in patients with more severe autonomic symptoms (97), which also appears to involve some contradictory aspects. Combined with its potential concentration-dependent role in neuroinflammation, this suggests during the active phase of CH, S100B is released by activated neuroglial cells, both triggering and sustaining inflammatory processes. However, under prolonged inflammatory conditions, excessive S100B consumption may occur, and such depletion could activate a compensatory negative feedback loop, thereby suppressing the subsequent release of inflammatory mediators. Notably, this just represents one potential mechanism underlying the observed changes. The bidirectional phenotypic transition of glial cells between pro-inflammatory and anti-inflammatory states across different CH stages may also play a role in this process. Therefore, further research is needed to elucidate the specific regulatory mechanisms involved in this process.

#### 4.1.3 Other pro-inflammatory cytokines

In an animal study, interleukin-6 (IL-6) in the rat dura mater has been shown to induce abnormal facial pain when exposed to subthreshold stimuli or even without overt stimulation (98),which suggests that IL-6 might modulate pain pathways by lowering the threshold for pain perception. In CH patients, plasma levels of the inflammatory factor IL-6 were found to be elevated during the attack phase, as indicated by antibody microarray analysis (99). Furthermore, the pro-inflammatory substance nitric oxide (NO) donors mediate a bidirectional regulation of IL-6 levels through NF-κB (100). It has been hypothesized that IL-6 may act by promoting the release of NO or by acting directly as a pain-causing component; however, more evidence is needed to support this hypothesis. An earlier study (101)found that S100B induced the release of IL-6 in neurons, and it remains to be investigated whether a correlation exists between the two in the inflammatory response associated with CH. Additionally, histamine release and leukotriene production in plasma during remission of CH (102), as well as elevated IL-2 expression during CH exacerbations (103), have also been reported. However, the specific mechanisms underlying these observations remain unclear due to the limited number of related studies.

While current inferences partially clarify the neuroinflammatory mechanisms in CH, targeted gene knockout combined or pharmacological inhibition of pro-inflammatory cytokines and their pathway-associated receptors remains necessary to validate causal relationships across distinct pathological stages. Currently, most research on cytokines related to primary headaches has focused on migraine and tension-type headaches, with a lack of effective clinical studies on CH (104). Various factors, including the site of blood collection, different sample analyses, and the individual condition of the patient, may influence cytokine levels to differing extents. Therefore, further large-scale clinical studies will be essential for a more comprehensive understanding of the inflammatory cytokines’ pathological mechanisms in CH neuroinflammation.

### 4.2 Nitric oxide

Nitric oxide (NO), a crucial neuromodulator, plays a significant role in regulating neuronal excitability and neurotransmitter release, while its vasodilatory effects are integral to various physiological mechanisms, including neuroinflammation (105). Early animal studies have demonstrated that inhibition of NO synthesis markedly decreases the activity of trigeminal cervical neurons, thereby obstructing the release of vasodilators (106). Conversely, the release of NO may provide a pathophysiological basis for the development of recurrent headaches. Additionally, NO is believed to facilitate the transmission of nociceptive signals from peripheral to central nervous systems and to sustain central nociceptive sensitization (107, 108).

NO donor triglycerides have emerged as a reliable model for inducing CH (109). Nitric oxide synthase (NOS) catalyzes the conversion of L-arginine to L-citrulline, a critical step in the production of NO. Notably, serum levels of L-arginine and citrulline are diminished in patients with chronic CH, suggesting an increased consumption rate for the synthesis and release of NO (110, 111). Consequently, it can be inferred that the repeated release of NO leads to trigeminal vasodilation, which contributes to the mechanisms underlying recurrent episodes of chronic CH. Furthermore, plasma levels of the NO metabolites nitrite (NO2-) and L-citrulline during periods without CH episodes do not significantly differ from those of healthy controls (112). However, measurements of NO2- and nitrate in cerebrospinal fluid during active episodes versus remission periods reveal an elevated concentration of total NOx (113). Despite the differing conclusions of various studies, it can be inferred that trigeminal nervous system sensitization occurs only when a specific threshold of NO release is surpassed, which subsequently plays a role in the vasodilatory and neurogenic inflammatory responses.

NOS comprises three subtypes: neurogenic (nNOS), endothelial (eNOS), and inducible (iNOS) (114). Among these, nNOS and eNOS are predominantly found in neural tissues. Experiments conducted on mice have demonstrated that both CGRP and NOS receptor blockers can inhibit nitroglycerin-induced neuronal activation (115). The increased activity of nNOS in trigeminal ganglion neurons leads to heightened production of NO, which subsequently induces the release of CGRP. This release of CGRP further mediates the production and release of NO through the activation of eNOS, resulting in the relaxation of vascular smooth muscle and subsequent vasodilation (116). NO and CGRP synergistically interact and may also contribute to the generation of neuroinflammation via this pathway; however, there have been no relevant clinical studies on CH. Although the iNOS gene test in CH yielded negative results, the combination of its variants may elevate the risk of developing CH (117).

## 5 Potential interactions of major molecular mechanisms

In animal models, researchers have demonstrated that chemical stimulation of the trigeminal nerve results in a significant increase in the release of CGRP and S100B within the targeted area, along with a marked upregulation of S100B expression in glial cells outside the stimulated region (89). Whereas injection of CGRP into the trigeminal ganglion results in increased release of glial fibrillary acidic protein (GFAP), a marker of glial cells (118). These findings suggest that activation of trigeminal neurons may elicit a reactive response in glial cells. Astrocytes and microglia are capable of recognizing inflammatory responses and producing inflammatory cytokines, such as IL-1β, IL-6, and various chemokines, to mediate neuroinflammation (119, 120). Moreover, IL-1β has been shown to promote astrocyte proliferation in a time- and dose-dependent manner; this activation cascade might further trigger the activation of local neurons, potentially contributing to the long-term maintenance of chronic inflammatory pain (121, 122). Some investigators have reported that the injection of CGRP into the trigeminal ganglion stimulates an increase in IL-1β mRNA expression in purified glial cells, a change that can be reversed by the neuroglial cell inhibitor minocycline, concurrently with an alleviation of CGRP-induced pain sensitivity (118). This observation implies that glial cells may be involved in CGRP-mediated inflammatory responses and facial pain transmission. Another study found that CGRP stimulation drives the inflammatory response by regulating inflammatory genes such as IL-1 in trigeminal glial cells and that the NF-κB pathway can act as one of the pathways to mediate this process (123). In summary, the converging evidence indicates that signal transduction between glial cells and neurons in the trigeminal nerve may operate within a positive feedback loop, and this process could involve glial cell-mediated inflammatory responses with CGRP acting as a key mediator.

**Figure 1:**
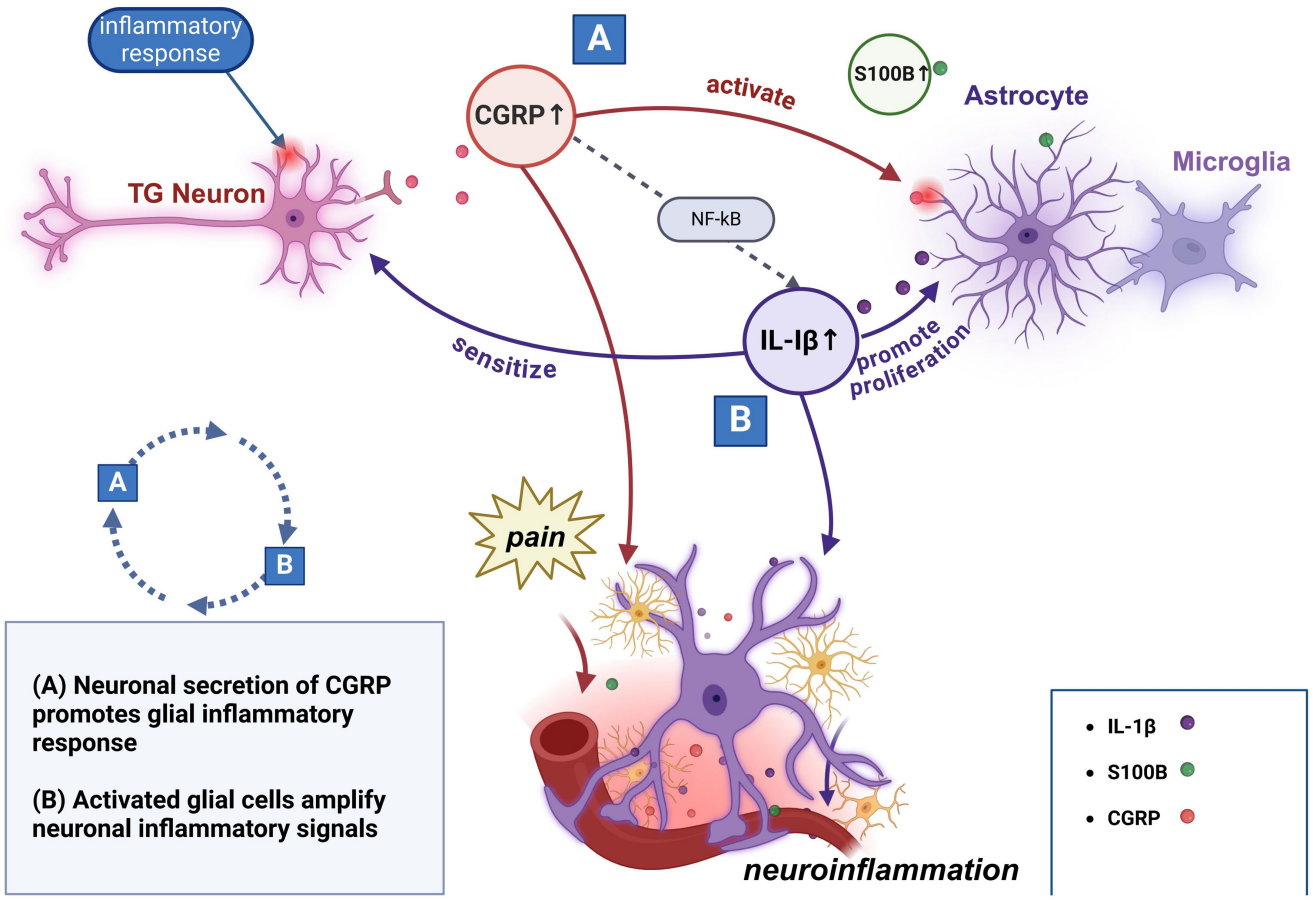
Potential interactions of primary molecular mechanisms in neuroinflammation.

Given the similarities between relevant mediators alterations observed in preclinical studies and observations in clinical studies of CH, it is hypothesized that this mechanism also occurs during neuroinflammation in CH. Specifically, activation of the trigeminal nerve leads to the release of CGRP, which not only directly participates in the inflammatory process and promotes pain transmission, but also activates neighboring glial cells to secrete a variety of inflammatory mediators, including IL-1β, IL-6, and S100B. This process may then establish a positive feedback loop that further enhances neuronal activation and prolongs the pain and inflammatory response (Figure 1).

Furthermore, it is important to note in particular that this inference has inherent limitations. Firstly, most of the existing studies are based on animal models and *in vitro* experiments, and there is a relative lack of clinical studies on CH patients to verify their relevance. Seccondly, episodes in CH patients involve cyclic transitions between attack and remission phases, which may place special demands on the dynamic regulation of positive feedback loops. In addition, the formation of the positive feedback loop is not the result of the action of a single factor but may involve complex multifactorial interactions. It remains unclear whether other inflammatory mediators and their interactions with glial cells might play a more significant role in this possible mechanism.

A recent study summarizes that multiple inflammatory cytokines and chemokines are upregulated in migraines, acting as mediators between glial cells and neurons to participate in inflammatory responses and pain transmission, while specific receptors and signaling pathways (e.g., NF-κB, MAPK) play an important role in glial cell activation and neuronal hypersensitivity (124). Although similar studies have not yet been conducted in CH, based on the fact that both headache types involve the activation of trigeminal neurons and multiple inflammatory mediators, glial cell-neuron interactions and their regulatory molecular mechanisms may provide new ideas for the study of the pathomechanisms of CH. In contrast to the strategy of blocking the CGRP pathway, it is worth considering whether limiting the transition of glial cells toward a pro-inflammatory phenotype or targeting specific receptors expressed on these cells could mitigate or disrupt this positive feedback loop, thereby controlling the occurrence of CH attacks, which also offers potential directions for future research.

## 6 Neuroinflammatory imaging evidence

Neuroinflammation can be observed in the early and prodromal stages of neurological diseases. Magnetic resonance imaging (MRI) is effective in revealing various biophysical tissue properties that are closely associated with the neuroinflammatory process (125), and it has been widely utilized in the pathophysiological investigation of inflammation-related neurological disorders (126, 127). Local cerebral blood flow (CBF) and CGRP release were increased after stimulation of the trigeminal ganglion in cats (128), suggesting that central pain mechanisms are involved in the pathogenesis of CH (129). MRI and positron emission computed tomography (PET) have confirmed the activation of multiple brain regions, including the hypothalamus and midbrain dopamine circuits, in the pathogenesis of CH (130, 131). However, there has been limited direct imaging evidence linking CH with neuroinflammation.

In migraine patients, neuroimaging techniques can serve as evidence of neuroinflammatory involvement by helping to identify vascular permeability and inflammatory cell activity (132). In migraine patients, high-resolution techniques such as 7 T MRI combined with TSPO-PET have demonstrated white matter microstructural abnormalities in the trigeminal root region, increased vascular permeability, and elevated TSPO signals that correlate with local microglial activation and suppressed function of the spinal trigeminal nucleus (133). These findings suggest, from an imaging perspective, that activation of the trigeminal vascular pathway during migraine attacks is associated with implied neuroinflammation. Based on the overlapping pathophysiologic mechanisms of the two disorders, the increase in neuroinflammatory markers in migraine justifies the need for multimodal imaging studies of CH. Future studies using advanced imaging modalities may be able to further elucidate whether similar patterns of microglia activation and vascular permeability are present in patients with CH, which could not only provide additional corroborating evidence to clarify its pathogenesis, but also advance the development of anti-inflammatory therapies targeting shared pathways in these headache disorders.

## 7 Future prospects

### 7.1 Shared pathological mechanisms between migraine and CH

Despite the clinical phenotypic differences between migraine and CH, they may share some pathophysiological mechanisms involving trigeminovascular activation and an underlying neuroinflammatory response (134). The involvement of vasodilation, cytokine dynamics, pro-inflammatory neuropeptide release, and glial cell activation in the neuroinflammatory mechanisms of migraine informs the understanding of the pathophysiology of CH, and could inform therapeutic strategies targeting inflammatory mediators. In addition, parasympathetic pathway activation is closely associated with intracranial autonomic symptoms triggered by headache attacks, exacerbating trigeminal sensitization through vascular dilatation and inflammatory amplification, a mechanism particularly prominent in CH and may be a specific therapeutic target. Notably, the cyclical nature of CH attacks implies unique molecular regulatory networks distinct from migraine, highlighting the need to balance the exploration of common mechanisms with disease-specific pathology.

### 7.2 Advancing from molecular mechanisms to clinical implementation

At the molecular level, further elucidation of the specific mechanisms by which inflammation triggers CH attacks remains a priority for future research. First, future studies should focus on the dynamic evolution of the CH inflammatory response during both the onset and remission phases, and analyze how temporal changes in inflammation affect the severity and duration of CH attacks, thereby providing a basis for phased intervention. Second, it is necessary to elucidate the signaling pathways through which different inflammatory mediators and key neuropeptides participate in the neuroinflammatory process of CH, including both their independent roles (e.g., the direct activation of parasympathetic pathways by neuropeptides) and their synergistic mechanisms (e.g., the combined activation of the trigeminal vascular system by inflammatory cytokines and neuropeptides). In addition, it is worth exploring whether various relevant mediators are involved in the disease process as triggers or maintenance factors during CH attacks.

Only by clarifying the molecular mechanisms of CH neuroinflammation and tracking the dynamic changes of inflammation can we provide accurate guidance for clinical application. Some literature reviews have compiled research on therapeutic mechanisms across different stages of CH (135, 136). Analysis of existing studies demonstrates that some mechanisms involving neuroinflammation may be partially linked to CGRP inhibition. While corticosteroids and triptans also likely function by suppressing pro-inflammatory factors and related signaling pathways. Additionally, lithium (a prophylactic agent) may relieve pain through inhibition of VIP-induced vasodilation. Available evidence suggests that the pathogenesis of CH may involve multiple molecules within the inflammatory cascade. Thus, a combined approach that targets both inflammatory mediator pathways (e.g., IL-1β) and neuropeptide signaling (e.g., CGRP) may hold greater promise in the future. Meanwhile, the continuous development of related technologies has significantly advanced disease research. The integration of gene editing, molecular testing, and imaging facilitates the identification of new molecular mechanisms in inflammatory responses and the detection of dynamic changes in inflammatory mediators at different stages of the disease, while multi-omics technologies help resolve the dynamic interactions among different inflammatory pathways. It is believed that, in the future, the use of these technologies as auxiliary tools in CH research will further facilitate the translation of molecular mechanisms into clinical applications.

## 8 Conclusion

The latest guidelines issued by the European Academy of Neurology recommend the use of 100% oxygen and subcutaneous sumatriptan during the acute phase, corticosteroids in the transitional phase, and oral verapamil for prophylaxis (137). However, the efficacy and risks associated with all existing treatments remain questionable (138). Although the pathogenesis of CH has not yet been fully elucidated, neuroinflammation is believed to play a pivotal role, potentially serving as both a trigger for CH and a key factor in its persistence and progression. A variety of inflammatory mediators and neuropeptides (Table 1) are collectively involved in neurogenic inflammation in CH, either through independent pathways or in conjunction with one another. Research focusing on these inflammatory mechanisms can enhance our understanding of the pathophysiological processes underlying CH and identify new, precise treatment targets. Furthermore, this approach may facilitate the development of personalized treatments for CH, tailored to different inflammatory response patterns and associated gene expression. The current lack of clinical understanding of CH is complicated by various factors that interfere with accurate diagnosis (139). Additionally, the transient nature of CH episodes, combined with insufficient preclinical studies and small sample sizes, poses significant challenges to advancing our understanding of the disease’s pathophysiological mechanisms. We anticipate that forthcoming research findings will yield new benefits for patients.

**Table 1:** Neuropeptides and inflammatory mediators in CH.

Mediator	Some findings	Evidence limitations
CGRP	- Elevated in jugular blood during CH attacks (43) - Decreased after triptans use during attacks (46) - Monoclonal antibody Galcanezumab partially effective in ECH; ineffective in CCH (51–53)	- Unclear temporal relationship with attack onset - Methodological variations across studies
PACAP-38 and VIP	- Elevated plasma concentrations during CH attacks (43, 74) - Sumatriptan partially reduces PACAP-38 activation (73) - Unchanged after CGRP infusion (44) - Associated with sensitization of neurons	- Smaller evidence base compared to CGRP - Unclear role in attack initiation versus propagation - Few studies differentiating effects of PACAP-38 vs. VIP
IL-1β	- MPD could suppresses expression (86) -Elevated in serum levels during CH attacks (87) - Contributes to glial activation and pro-inflammatory cascade (121, 122) - A feedback mechanism may exist with CGRP (88, 89)	- Inconsistent findings across different studies - Limited number of human studies - Small sample sizes in available studies
S100B and NF-κB Pathway	- Elevated levels during CH attacks (96, 97) - S100B is decreased in patients with multiple autonomic symptoms (97) - Mediates inflammatory cytokines expression in Glia cells - Promotes glial-neuronal interaction	- Few CH-specific studies available - Findings mostly derived from migraine or preclinical models - Unclear causal relationship with CH pathophysiology
NO	- Contributes to vasodilation (106) - NO donors can trigger CH attacks (109) - Interacts with CGRP release mechanisms (116)	- Limited direct evidence specific to CH - Challenges in measuring NO directly in human studies

## References

[1] Olesen J. Headache classification Committee of the International Headache Society (Ihs) the international classification of headache disorders, 3rd Edition. Cephalalgia. (2018) 38:1–211. doi: 10.1177/0333102417738202 29368949

[2] Kim SA Choi SY Youn MS Pozo-Rosich P Lee MJ. Epidemiology, burden and clinical Spectrum of cluster headache: a global update. Cephalalgia. (2023) 43:43. doi: 10.1177/03331024231201577, PMID: 37728577

[3] Negro A Sciattella P Spuntarelli V Martelletti P Mennini FS. Direct and indirect costs of cluster headache: a prospective analysis in a tertiary level headache Centre. J Headache Pain. (2020) 21:44. doi: 10.1186/s10194-020-01115-4, PMID: 32366217 PMC7197153

[4] Kim B-S Chung P-W Kim B-K Lee MJ Park JW Chu MK. The impact of remission and coexisting migraine on anxiety and depression in cluster headache. J Headache Pain. (2020) 21:58. doi: 10.1186/s10194-020-01120-7, PMID: 32471362 PMC7257141

[5] Koo BB Bayoumi A Albanna A Abusuliman M Burrone L Sico JJ. Demoralization predicts suicidality in patients with cluster headache. J Headache Pain. (2021) 22:28. doi: 10.1186/s10194-021-01241-7, PMID: 33879041 PMC8056539

[6] Fox J Gaul C Slijepcevic M Ohse J Peperkorn N Shiban Y. The impact of fear of attacks on pain-related disability in cluster headache: insights from the fear avoidance model. Headache. (2024) 65:45–53. doi: 10.1111/head.14823, PMID: 39224926 PMC11725993

[7] Zhang SH Xu SY Chen CF Xue ZY Yao YR Zhao HR. Profile of Chinese cluster headache register individual study (Chris): clinical characteristics, diagnosis and treatment status data of 816 patients in China. Cephalalgia. (2024) 44:44. doi: 10.1177/03331024241235193, PMID: 38501875

[8] Membrilla JA Cuadrado ML González-García N Porta-Etessam J Sánchez-Soblechero A Ros AL. The profile of refractory chronic cluster headache. Neurol Sci. (2024) 46:295–302. doi: 10.1007/s10072-024-07708-0, PMID: 39044103

[9] Fischera M Marziniak M Gralow I Evers S. The incidence and prevalence of cluster headache: a Meta-analysis of population-based studies. Cephalalgia. (2008) 28:614–8. doi: 10.1111/j.1468-2982.2008.01592.x 18422717

[10] DiSabato DJ Quan N Godbout JP. Neuroinflammation: the devil is in the details. J Neurochem. (2016) 139:136–53. doi: 10.1111/jnc.13607, PMID: 26990767 PMC5025335

[11] Lyman M Lloyd DG Ji XM Vizcaychipi MP Ma DQ. Neuroinflammation: the role and consequences. Neurosci Res. (2014) 79:1–12. doi: 10.1016/j.neures.2013.10.004 24144733

[12] Ran C Olofsgard FJ Wellfelt K Steinberg A Belin AC. Elevated cytokine levels in the central nervous system of cluster headache patients in bout and in remission. J Headache Pain. (2024) 25:121. doi: 10.1186/s10194-024-01829-9, PMID: 39044165 PMC11267889

[13] Brack A Rittner HL Stein C. Neurogenic Painful Inflammation. Curr Opin Anaesthesiol. (2004) 17:461–4. doi: 10.1097/00001503-200410000-00018, PMID: 17023906

[14] Guan LC Dong XZ Green DP. Roles of mast cells and their interactions with the trigeminal nerve in migraine headache. Mol Pain. (2023) 19:19. doi: 10.1177/17448069231181358, PMID: 37232078 PMC10262643

[15] Spekker E Tanaka M Szabó Á Vécsei L. Neurogenic inflammation: the participant in migraine and recent advancements in translational research. Biomedicines. (2021) 10:10. doi: 10.3390/biomedicines10010076, PMID: 35052756 PMC8773152

[16] Akerman S Romero-Reyes M Holland PR. Current and novel insights into the neurophysiology of migraine and its implications for therapeutics. Pharmacol Ther. (2017) 172:151–70. doi: 10.1016/j.pharmthera.2016.12.005, PMID: 27919795

[17] Hoffmann J Baca SM Akerman S. Neurovascular mechanisms of migraine and cluster headache. J Cereb Blood Flow Metab. (2019) 39:573–94. doi: 10.1177/0271678X17733655, PMID: 28948863 PMC6446418

[18] Akerman S Holland PR Summ O Lasalandra MP Goadsby PJ. A translational in vivo model of trigeminal autonomic Cephalalgias: therapeutic characterization. Brain. (2012) 135:3664–75. doi: 10.1093/brain/aws249, PMID: 23065481

[19] Wu JW Chen ST Wang YF Chen SP Tseng SY Kuo YS. Pre-cluster symptoms in a Taiwanese cohort of cluster headache: symptom profiles and clinical predictions. J Headache Pain. (2024) 25:174. doi: 10.1186/s10194-024-01862-8, PMID: 39379823 PMC11460087

[20] Petersen AS Lund N Goadsby PJ Belin AC Wang S-J Fronczek R. Recent advances in diagnosing, managing, and understanding the pathophysiology of cluster headache. Lancet Neurol. (2024) 23:712–24. doi: 10.1016/S1474-4422(24)00143-1, PMID: 38876749

[21] Penfield W McNaughton F. Dural headache and innervation of the dura mater. J Nerv Ment Dis. (1941) 94:748. doi: 10.1097/00005053-194112000-00023

[22] Marfurt CF. The central projections of trigeminal primary afferent neurons in the cat as determined by the Tranganglionic transport of horseradish peroxidase. J Comp Neurol. (2004) 203:785–98. doi: 10.1002/cne.902030414, PMID: 6173403

[23] Chen S-P Takizawa T Sekiguchi K Nakahara J Wang S-J. Covid-19 vaccination elicited De novo and recurrence of cluster headache: a case series. Cephalalgia. (2023) 43:43. doi: 10.1177/03331024231173354, PMID: 37138462

[24] Aşkın Turan S Aydın Ş. A retrospective cohort study: is Covid-19 Bnt162b2 Mrna vaccination a trigger factor for cluster headache? Acta Neurol Belg. (2024) 124:1535–42. doi: 10.1007/s13760-024-02536-7, PMID: 38619748

[25] Brandt RB Ouwehand RLH Ferrari MD Haan J Fronczek R. Covid-19 vaccination-triggered cluster headache episodes with frequent attacks. Cephalalgia. (2022) 42:1420–4. doi: 10.1177/03331024221113207, PMID: 35833226 PMC9638705

[26] Chen S-P Hsu C-L Wang Y-F Yang F-C Chen T-H Huang J-H. Genome-wide analyses identify novel risk loci for cluster headache in Han Chinese residing in Taiwan. J Headache Pain. (2022) 23:147. doi: 10.1186/s10194-022-01517-6, PMID: 36404298 PMC9677903

[27] Ji RR Nackley A Huh Y Terrando N Maixner W. Neuroinflammation and central sensitization in chronic and widespread pain. Anesthesiology. (2018) 129:343–66. doi: 10.1097/ALN.0000000000002130, PMID: 29462012 PMC6051899

[28] Vergne-Salle P Bertin P. Chronic pain and Neuroinflammation. Joint Bone Spine. (2021) 88:105222. doi: 10.1016/j.jbspin.2021.105222, PMID: 34022418

[29] Buture A Boland JW Dikomitis L Ahmed F. Update on the pathophysiology of cluster headache: imaging and neuropeptide studies. J Pain Res. (2019) 12:269–81. doi: 10.2147/JPR.S175312, PMID: 30655693 PMC6324919

[30] Schuh-Hofer S Siekmann W Offenhauser N Reuter U Arnold G. Effect of Hyperoxia on neurogenic plasma protein extravasation in the rat dura mater. Headache. (2006) 46:1545–51. doi: 10.1111/j.1526-4610.2006.00447.x, PMID: 17115987

[31] Merli E Rustici A Gramegna LL Di Donato M Agati R Tonon C. Vessel-Wall Mri in primary headaches: the role of neurogenic inflammation. Headache. (2022) 63:1372–9. doi: 10.1111/head.14253 35137395

[32] Messlinger K. The big Cgrp flood - sources, sinks and Signalling sites in the Trigeminovascular system. J Headache Pain. (2018) 19:22. doi: 10.1186/s10194-018-0848-0, PMID: 29532195 PMC5847494

[33] Storer RJ Akerman S Goadsby PJ. Calcitonin gene-related peptide (Cgrp) modulates nociceptive Trigeminovascular transmission in the cat. Br J Pharmacol. (2004) 142:1171–81. doi: 10.1038/sj.bjp.0705807, PMID: 15237097 PMC1575174

[34] Cady RJ Glenn JR Smith KM Durham PL. Calcitonin gene-related peptide promotes cellular changes in trigeminal neurons and glia implicated in peripheral and central sensitization. Mol Pain. (2011) 7:94. doi: 10.1186/1744-8069-7-94, PMID: 22145886 PMC3267674

[35] Manganotti P Deodato M D’Acunto L Biaduzzini F Garascia G Granato A. Effects of anti-Cgrp monoclonal antibodies on neurophysiological and clinical outcomes: a combined transcranial magnetic stimulation and Algometer study. Neurol Int. (2024) 16:673–88. doi: 10.3390/neurolint16040051, PMID: 39051212 PMC11270432

[36] Xie S Gao ZF Zhang JL Xing C Dong YX Wang LY. Monoclonal antibody targeting Cgrp relieves cisplatin-induced neuropathic pain by attenuating Neuroinflammation. Neurotox Res. (2024) 42:8. doi: 10.1007/s12640-023-00685-w, PMID: 38194189

[37] Wu W Feng BS Liu J Li Y Liao Y Wang SQ. The Cgrp/macrophage Axis signal facilitates inflammation recovery in the intestine. Clin Immunol. (2022) 245:245. doi: 10.1016/j.clim.2022.109154, PMID: 36243345

[38] Glowka TR Steinebach A Stein K Schwandt T Lysson M Holzmann B. The novel Cgrp receptor antagonist Bibn4096bs alleviates a postoperative intestinal inflammation and prevents postoperative ileus. Neurogastroenterol Motil. (2015) 27:1038–49. doi: 10.1111/nmo.12584 25929169

[39] Dakhama A. Calcitonin gene-related peptide: role in airway homeostasis. Curr Opin Pharmacol. (2004) 4:215–20. doi: 10.1016/j.coph.2004.01.006, PMID: 15140411

[40] Li J Vause CV Durham PL. Calcitonin gene-related peptide stimulation of nitric oxide synthesis and release from trigeminal ganglion glial cells. Brain Res. (2008) 1196:22–32. doi: 10.1016/j.brainres.2007.12.028, PMID: 18221935 PMC2268710

[41] Sacco S Bendtsen L Ashina M Reuter U Terwindt G Mitsikostas D-D. European headache federation guideline on the use of monoclonal antibodies acting on the calcitonin gene related peptide or its receptor for migraine prevention. J Headache Pain. (2019) 20:6. doi: 10.1186/s10194-018-0955-y, PMID: 30651064 PMC6734227

[42] Moreno-Ajona D Pérez-Rodríguez A Goadsby PJ. Gepants, calcitonin-gene-related peptide receptor antagonists: what could be their role in migraine treatment? Curr Opin Neurol. (2020) 33:309–15. doi: 10.1097/WCO.0000000000000806, PMID: 32251023

[43] Goadsby PJ Edvinsson L. Human in vivo evidence for Trigeminovascular activation in cluster headache neuropeptide changes and effects of acute attacks therapies. Brain. (1994) 117:427–34. doi: 10.1093/brain/117.3.427, PMID: 7518321

[44] Snoer A Vollesen ALH Beske RP Guo S Hoffmann J Fahrenkrug J. Calcitonin gene-related peptide and disease activity in cluster headache. Cephalalgia. (2019) 39:575–84. doi: 10.1177/0333102419837154, PMID: 30854880

[45] Petersen AS Lund N Messlinger K Christensen SL Barloese M Jorgensen NR. Reduced plasma calcitonin gene-related peptide level identified in cluster headache: a prospective and controlled study. Cephalalgia. (2024) 44:1–11. doi: 10.1177/03331024231223970, PMID: 38436282

[46] Kamm K Straube A Ruscheweyh R. Baseline tear fluid Cgrp is elevated in active cluster headache patients as long as they have not taken attack abortive medication. Cephalalgia. (2020) 41:69–77. doi: 10.1177/0333102420949858, PMID: 32847402

[47] Stepień A Jagustyn P Trafny EA Widerkiewicz K. Suppressing effect of the serotonin 5ht1b/D receptor agonist Rizatriptan on calcitonin gene-related peptide (Cgrp) concentration in migraine attacks. Neurol Neurochir Pol. (2003) 37:1013–23. Available at: https://pubmed.ncbi.nlm.nih.gov/15174248/ PMID: 15174248

[48] Benemei S Cortese F Labastida-Ramírez A Marchese F Pellesi L Romoli M. Triptans and Cgrp blockade – impact on the cranial vasculature. J Headache Pain. (2017) 18:103. doi: 10.1186/s10194-017-0811-5, PMID: 29019093 PMC5635141

[49] Vollesen ALH Snoer A Beske RP Guo S Hoffmann J Jensen RH. Effect of infusion of calcitonin gene-related peptide on cluster headache attacks a randomized clinical trial. JAMA Neurol. (2018) 75:1187–97. doi: 10.1001/jamaneurol.2018.1675, PMID: 29987329 PMC6233850

[50] Mudugal D Monteith TS. Drug profile: Galcanezumab for prevention of cluster headache. Expert Rev Neurother. (2021) 21:145–55. doi: 10.1080/14737175.2021.1852931, PMID: 33206562

[51] Pellesi L De Icco R Al-Karagholi MA-M Ashina M. Reducing episodic cluster headaches: focus on Galcanezumab. J Pain Res. (2020) 13:1591–9. doi: 10.2147/JPR.S222604, PMID: 32753938 PMC7342329

[52] Chen ST Wu JW. Cgrp-targeted therapy for episodic and chronic cluster headache. Curr Pain Headache Rep. (2022) 26:667–75. doi: 10.1007/s11916-022-01070-6, PMID: 35881279

[53] Chan C Goadsby PJ. Cgrp pathway monoclonal antibodies for cluster headache. Expert Opin Biol Ther. (2020) 20:947–53. doi: 10.1080/14712598.2020.1751114, PMID: 32241175

[54] Goadsby PJ Dodick DW Leone M Bardos JN Oakes TM Millen BA. Trial of Galcanezumab in prevention of episodic cluster headache. N Engl J Med. (2019) 381:132–41. doi: 10.1056/NEJMoa1813440 31291515

[55] Hong Y Kang MK Moon HS Kim BK Cho SJ. Preventive therapy with Galcanezumab for two consecutive cluster bouts in patients with episodic cluster headache: An observational multicenter study. J Headache Pain. (2023) 24:1661. doi: 10.1186/s10194-023-01661-7, PMID: 37817084 PMC10566025

[56] Riesenberg R Gaul C Stroud CE Dong Y Bangs ME Wenzel R. Long-term open-label safety study of Galcanezumab in patients with episodic or chronic cluster headache. Cephalalgia. (2022) 42:1225–35. doi: 10.1177/03331024221103509, PMID: 35633025

[57] Dodick DW Goadsby PJ Lucas C Jensen R Bardos JN Martinez JM. Phase 3 randomized, placebo-controlled study of Galcanezumab in patients with chronic cluster headache: results from 3-month double-blind treatment. Cephalalgia. (2020) 40:935–48. doi: 10.1177/0333102420905321, PMID: 32050782 PMC7787002

[58] Ruscheweyh R Broessner G Goßrau G Heinze-Kuhn K Jürgens TP Kaltseis K. Effect of calcitonin gene-related peptide (-receptor) antibodies in chronic cluster headache: results from a retrospective case series support individual treatment attempts. Cephalalgia. (2020) 40:1574–84. doi: 10.1177/0333102420949866, PMID: 32806953 PMC7691634

[59] Medrea I Christie S Tepper SJ Thavorn K Hutton B. Effects of acute and preventive therapies for episodic and chronic cluster headache: a scoping review of the literature. Headache. (2022) 62:329–62. doi: 10.1111/head.14284, PMID: 35315067

[60] Goadsby PJ Barbanti P Lambru G Ettrup A Christoffersen CL Josiassen MK. Eptinezumab improved patient-reported outcomes and quality of life in patients with migraine and prior preventive treatment failures. Eur J Neurol. (2023) 30:1089–98. doi: 10.1111/ene.15670 36583633

[61] Simmonds L Jamtøy KA Aschehoug I Hara S Meisingset TW Matharu MS. Open label experience of repeated Onabotulinumtoxina injections towards the sphenopalatine ganglion in patients with chronic cluster headache and chronic migraine. Cephalalgia. (2024) 44:44. doi: 10.1177/03331024241273967, PMID: 39165124

[62] Kollenburg L Arnts H Heitkamp M Geerts S Robinson C Dominguez M. Occipital nerve stimulation for cluster headache: lessons to learn from the ‘voltage tuners’. J Headache Pain. (2024) 25:139. doi: 10.1186/s10194-024-01839-7, PMID: 39180011 PMC11344319

[63] Lansbergen CS de Vos CC Brandt RB Ferrari MD Huygen FJPM Fronczek R. Occipital nerve stimulation in medically intractable chronic cluster headache. Eur J Neurol. (2024) 31:e16212. doi: 10.1111/ene.16212, PMID: 38230580 PMC11235842

[64] Membrilla JA Cuadrado ML Gonzalez-Garcia N Porta-Etessam J Sanchez-Soblechero A Lozano-Ros A. Clinical predictors of therapeutic failure of occipital nerve stimulation in refractory chronic cluster headache. J Headache Pain. (2024) 44:44. doi: 10.1177/03331024241254078, PMID: 38825586

[65] Lamas Pérez R Millán-Vázquez M González-Oria C. Efficacy and safety of Galcanezumab as chronic cluster headache preventive treatment under real world conditions: observational prospective study. Cephalalgia. (2024) 44:44. doi: 10.1177/03331024231226181, PMID: 38501892

[66] Sándor K Kormos V Botz B Imreh A Bölcskei K Gaszner B. Impaired Nocifensive Behaviours and mechanical hyperalgesia, but enhanced thermal allodynia in pituitary adenylate cyclase-activating polypeptide deficient mice. Neuropeptides. (2010) 44:363–71. doi: 10.1016/j.npep.2010.06.004, PMID: 20621353

[67] Ohnou T Yokai M Kurihara T Hasegawa-Moriyama M Shimizu T Inoue K. Pituitary adenylate cyclase-activating polypeptide type 1 receptor signaling evokes long-lasting nociceptive behaviors through the activation of spinal astrocytes in mice. J Pharmacol Sci. (2016) 130:194–203. doi: 10.1016/j.jphs.2016.01.008 26948958

[68] Vaudry D Gonzalez BJ Basille M Yon L Fournier A Vaudry H. Pituitary adenylate cyclase-activating polypeptide and its receptors: from structure to functions. Pharmacol Rev. (2000) 52:269–324. doi: 10.1016/S0031-6997(24)01449-2, PMID: 10835102

[69] Goadsby PJ. Autonomic nervous system control of the cerebral circulation. Handb Clin Neurol. (2013) 117:193–201. doi: 10.1016/B978-0-444-53491-0.00016-X, PMID: 24095126

[70] Wilkins BW Chung LH Tublitz NJ Wong BJ Minson CT. Mechanisms of vasoactive intestinal peptide-mediated vasodilation in human skin. J Appl Physiol. (2004) 97:1291–8. doi: 10.1152/japplphysiol.00366.2004, PMID: 15155712

[71] Frederiksen SD Warfvinge K Ohlsson L Edvinsson L. Expression of pituitary adenylate cyclase-activating peptide, calcitonin gene-related peptide and headache targets in the trigeminal ganglia of rats and humans. Neuroscience. (2018) 393:319–32. doi: 10.1016/j.neuroscience.2018.10.004, PMID: 30336190

[72] Akerman S Goadsby PJ. Neuronal Pac1 receptors mediate delayed activation and sensitization of Trigeminocervical neurons: relevance to migraine. Sci Transl Med. (2015) 7:308ra157. doi: 10.1126/scitranslmed.aaa7557, PMID: 26446954

[73] Akerman S Goadsby PJ Romero-Reyes M. Pacap-38 related modulation of the cranial parasympathetic projection: a novel mechanism and therapeutic target in severe primary headache. Br J Pharmacol. (2023) 181:480–94. doi: 10.1111/bph.16242, PMID: 37706270

[74] Tuka B Szabó N Tóth E Kincses ZT Párdutz Á Szok D. Release of Pacap-38 in episodic cluster headache patients – an exploratory study. J Headache Pain. (2016) 17:69. doi: 10.1186/s10194-016-0660-7, PMID: 27475101 PMC4967416

[75] Vollesen ALH Snoer A Chaudhry B Petersen AS Hagedorn A Hoffmann J. The effect of pituitary adenylate cyclase-activating Peptide-38 and vasoactive intestinal peptide in cluster headache. Cephalalgia. (2020) 40:1474–88. doi: 10.1177/0333102420940689, PMID: 32962406

[76] Deligianni C Pellesi L Chaudhry BA Haulund Vollesen AL Snoer AH Hannibal J. Plasma levels of Vip are not elevated during Pacap- and Vip-induced cluster headache attacks: An exploratory study. Front Neurol. (2023) 14:14. doi: 10.3389/fneur.2023.1135246, PMID: 37143998 PMC10151752

[77] Delgado M Jonakait GM Ganea D. Vasoactive intestinal peptide and pituitary adenylate cyclase-activating polypeptide inhibit chemokine production in activated microglia. Glia. (2002) 39:148–61. doi: 10.1002/glia.10098 12112366

[78] Pellesi L Chaudhry BA Vollesen ALH Snoer AH Baumann K Skov PS. Pacap38- and Vip-induced cluster headache attacks are not associated with changes of plasma Cgrp or markers of mast cell activation. Cephalalgia. (2021) 42:687–95. doi: 10.1177/03331024211056248, PMID: 34822741

[79] Edvinsson JCA Grell AS Warfvinge K Sheykhzade M Edvinsson L Haanes KA. Differences in pituitary adenylate cyclase-activating peptide and calcitonin gene-related peptide release in the Trigeminovascular system. Cephalalgia. (2020) 40:1296–309. doi: 10.1177/0333102420929026, PMID: 32486909

[80] Kuburas A Russo AF. Shared and independent roles of Cgrp and Pacap in migraine pathophysiology. J Headache Pain. (2023) 24:34. doi: 10.1186/s10194-023-01569-2, PMID: 37009867 PMC10069045

[81] Harmar AJ Fahrenkrug J Gozes I Laburthe M May V Pisegna JR. Pharmacology and functions of receptors for vasoactive intestinal peptide and pituitary adenylate cyclase-activating polypeptide: Iuphar review 1. Br J Pharmacol. (2012) 166:4–17. doi: 10.1111/j.1476-5381.2012.01871.x, PMID: 22289055 PMC3415633

[82] Hoffmann J Miller S Martins-Oliveira M Akerman S Supronsinchai W Sun H. Pac1 receptor blockade reduces central nociceptive activity: new approach for primary headache? Pain. (2020) 161:1670–81. doi: 10.1097/j.pain.0000000000001858, PMID: 32142016 PMC7302332

[83] Ashina M Doležil D Bonner JH Zhou L Klatt J Picard H. A phase 2, randomized, double-blind, placebo-controlled trial of Amg 301, a pituitary adenylate cyclase-activating polypeptide Pac1 receptor monoclonal antibody for migraine prevention. Cephalalgia. (2020) 41:33–44. doi: 10.1177/0333102420970889, PMID: 33231489 PMC7786389

[84] Guo S Rasmussen RH Hay-Schmidt A Ashina M Asuni AA Jensen JM. Vpac1 and Vpac2 receptors mediate tactile Hindpaw hypersensitivity and carotid artery dilatation induced by Pacap38 in a migraine relevant mouse model. J Headache Pain. (2024) 25:126. doi: 10.1186/s10194-024-01830-2, PMID: 39085771 PMC11293201

[85] Amantea D Bagetta G Tassorelli C Mercuri NB Corasaniti MT. Identification of distinct cellular pools of interleukin-1β during the evolution of the Neuroinflammatory response induced by transient middle cerebral artery occlusion in the Brain of rat. Brain Res. (2010) 1313:259–69. doi: 10.1016/j.brainres.2009.12.017, PMID: 20025855

[86] Neeb L Hellen P Hoffmann J Dirnagl U Reuter U. Methylprednisolone blocks interleukin 1 Beta induced calcitonin gene related peptide release in trigeminal ganglia cells. J Headache Pain. (2016) 17:19. doi: 10.1186/s10194-016-0609-x, PMID: 26931452 PMC4773314

[87] Martelletti P Granata M Giacovazzo M. Serum Interleukin-1 Beta is increased in cluster headache. Cephalalgia. (1993) 13:343–5. doi: 10.1046/j.1468-2982.1993.1305343.x, PMID: 7694804

[88] Neeb L Hellen P Boehnke C Hoffmann J Schuh-Hofer S Dirnagl U. Il-1β stimulates Cox-2 dependent Pge synthesis and Cgrp release in rat trigeminal ganglia cells. PLoS One. (2011) 6:e17360. doi: 10.1371/journal.pone.0017360, PMID: 21394197 PMC3048859

[89] Thalakoti S Patil VV Damodaram S Vause CV Langford LE Freeman SE. Neuron–glia signaling in trigeminal ganglion: implications for migraine pathology. Headache. (2007) 47:1008–23. doi: 10.1111/j.1526-4610.2007.00854.x, PMID: 17635592 PMC2268711

[90] Buckley TL Brain SD Collins PD Williams TJ. Inflammatory edema induced by interactions between Il-1 and the neuropeptide calcitonin gene-related peptide. J Immunol. (1991) 146:3424–30. doi: 10.4049/jimmunol.146.10.3424, PMID: 1673985

[91] Han DW. Association of Serum Levels of calcitonin gene-related peptide and cytokines during migraine attacks. Ann Indian Acad Neurol. (2019) 22:277–81. doi: 10.4103/aian.AIAN_371_18, PMID: 31359937 PMC6613407

[92] Yamanaka G Suzuki S Morishita N Takeshita M Kanou K Takamatsu T. Role of Neuroinflammation and blood-Brain barrier Permutability on migraine. Int J Mol Sci. (2021) 22:22. doi: 10.3390/ijms22168929, PMID: 34445635 PMC8396312

[93] Michetti F D'Ambrosi N Toesca A Puglisi MA Serrano A Marchese E. The S100b story: from biomarker to active factor in neural injury. J Neurochem. (2019) 148:168–87. doi: 10.1111/jnc.14574, PMID: 30144068

[94] Ben-Neriah Y Karin M. Inflammation meets Cancer, with Nf-Κb as the matchmaker. Nat Immunol. (2011) 12:715–23. doi: 10.1038/ni.2060, PMID: 21772280

[95] Michetti F Clementi ME Di Liddo R Valeriani F Ria F Rende M. The S100b protein: a multifaceted pathogenic factor more than a biomarker. Int J Mol Sci. (2023) 24:9605. doi: 10.3390/ijms24119605, PMID: 37298554 PMC10253509

[96] Snoer AH Vollesen ALH Beske RP Guo S Hoffmann J Jørgensen NR. S100b and Nse in cluster headache – evidence for glial cell activation? Headache. (2020) 60:1569–80. doi: 10.1111/head.13864, PMID: 32548854

[97] Şahin E Karaaslan Z Şanlı E Timirci Kahraman Ö Ulusoy C Kocasoy Orhan E. Reduced expression of Inflammasome complex components in cluster headache. Headache. (2022) 62:967–76. doi: 10.1111/head.14334, PMID: 35670197

[98] Burgos-Vega CC Quigley LD Avona A Price T Dussor G. Dural stimulation in rats causes Brain-derived neurotrophic factor-dependent priming to subthreshold stimuli including a migraine trigger. Pain. (2016) 157:2722–30. doi: 10.1097/j.pain.0000000000000692, PMID: 27841839 PMC5315498

[99] Shasha Z Xiaohui W Xiaolin W Shiwen W. Analysis of inflammatory cytokines in patients with cluster headache. Chin J Neuropsychiatr Dis. (2017) 43:274–8. doi: 10.3969/j.issn.1002-0152.2017.05.004

[100] Siednienko J Nowak J Moynagh PN Gorczyca WA. Nitric oxide affects Il-6 expression in human peripheral blood mononuclear cells involving Cgmp-dependent modulation of Nf-Κb activity. Cytokine. (2011) 54:282–8. doi: 10.1016/j.cyto.2011.02.015, PMID: 21414799

[101] Lybsllmrg WS. S100β induction of the Proinflammatory cytokine Interleukin-6 in neurons. J Neurochem. (2000) 74:143–50. doi: 10.1046/j.1471-4159.2000.0740143.x 10617115 PMC3836592

[102] Martelletti P Adriani E Bonini S Celestino D Lenti L Armaleo C. Basophil histamine release and leukotriene (Ltb4 - Ltc4) production in cluster headache. Headache. (1989) 29:46–8. doi: 10.1111/j.1526-4610.1989.hed2901046.x 2466813

[103] Steinberg A Sjöstrand C Sominanda A Fogdell-Hahn A AIM NR. Interleukin-2 gene expression in different phases of episodic cluster headache - a pilot study. Acta Neurol Scand. (2011) 124:130–4. doi: 10.1111/j.1600-0404.2010.01434.x 20880293

[104] Musubire AK Cheema S Ray JC Hutton EJ Matharu M. Cytokines in primary headache disorders: a systematic review and Meta-analysis. J Headache Pain. (2023):24. doi: 10.1186/s10194-023-01572-7 37016284 PMC10071234

[105] Ignarro LJ. Nitric oxide is not just blowing in the wind. Br J Pharmacol. (2018) 176:131–4. doi: 10.1111/bph.14540 PMC6295410 30556130

[106] Hoskin KL Bulmer DCE Goadsby PJ. Fos expression in the Trigeminocervical complex of the cat after stimulation of the superior sagittal sinus is reduced by L-name. Neurosci Lett. (1999) 266:173–6. doi: 10.1016/s0304-3940(99)00281-5 10465701

[107] Bagetta G Iannone M Del Duca C Nisticò G. Inhibition by Nω-nitro-L-arginine methyl Ester of the Electrocortical arousal response in rats. Br J Pharmacol. (1993) 108:858–60. doi: 10.1111/j.1476-5381.1993.tb13477.x 8485627 PMC1908148

[108] Zhang XC Kainz V Zhao J Strassman AM Levy D. Vascular extracellular signal-regulated kinase mediates migraine-related sensitization of meningeal nociceptors. Ann Neurol. (2013) 73:741–50. doi: 10.1002/ana.23873 23447360 PMC3688635

[109] Wei DY Goadsby PJ. Comprehensive clinical phenotyping of nitroglycerin infusion induced cluster headache attacks. Cephalalgia. (2021) 41:913–33. doi: 10.1177/0333102421989617 33615843 PMC8217894

[110] D’Andrea G Bussone G Di Fiore P Perini F Gucciardi A Bolner A. Pathogenesis of chronic cluster headache and bouts: role of tryptamine, arginine metabolism and Α1-agonists. Neurol Sci. (2017) 38:37–43. doi: 10.1007/s10072-017-2862-4 28527056

[111] D'Andrea G Gucciardi A Perini F Leon A. Pathogenesis of cluster headache: from episodic to chronic form, the role of neurotransmitters and neuromodulators. Headache. (2019) 59:1665–70. doi: 10.1111/head.13673 31603552

[112] Costa A Ravaglia S Sances G Antonaci F Pucci E Nappi G. Nitric oxide pathway and response to nitroglycerin in cluster headache patients: plasma nitrite and Citrulline levels. Cephalalgia. (2003) 23:407–13. doi: 10.1046/j.1468-2982.2003.00553.x 12807519

[113] Steinberg A Wiklund NP Brundin L Remahl AIMN. Levels of nitric oxide metabolites in cerebrospinal fluid in cluster headache. Cephalalgia. (2010) 30:696–702. doi: 10.1177/0333102409351799 20511209

[114] Fukuto JM Chaudhuri G. Inhibition of constitutive and inducible nitric oxide synthase: potential selective inhibition. Annu Rev Pharmacol Toxicol. (1995) 35:165–94. doi: 10.1146/annurev.pa.35.040195.001121 7541188

[115] Ramachandran R Bhatt DK Ploug KB Hay-Schmidt A Jansen-Olesen I Gupta S. Nitric oxide synthase, calcitonin gene-related peptide and Nk-1 receptor mechanisms are involved in Gtn-induced neuronal activation. Cephalalgia. (2014) 34:136–47. doi: 10.1177/0333102413502735 24000375

[116] Akerman S Williamson DJ Kaube H Goadsby PJ. Nitric oxide synthase inhibitors can antagonize neurogenic and calcitonin gene-related peptide induced dilation of Dural meningeal vessels. Br J Pharmacol. (2002) 137:62–8. doi: 10.1038/sj.bjp.0704842 12183331 PMC1573468

[117] Ran CE Michalska JM Fourier C Sjöstrand C Waldenlind E Steinberg A. Analysis of Nos gene polymorphisms in relation to cluster headache and predisposing factors in Sweden. Brain Sci. (2021):11. doi: 10.3390/brainsci11010034 33396232 PMC7824326

[118] Afroz S Arakaki R Iwasa T Oshima M Hosoki M Inoue M. Cgrp induces differential regulation of cytokines from satellite glial cells in trigeminal ganglia and orofacial nociception. Int J Mol Sci. (2019):20. doi: 10.3390/ijms20030711 30736422 PMC6386987

[119] Shastri A Bonifati DM Kishore U. Innate immunity and Neuroinflammation. Mediat Inflamm. (2013) 2013:1–19. doi: 10.1155/2013/342931 PMC3697414 23843682

[120] Tewari M Michalski S Egan TM. Modulation of microglial function by Atp-gated P2x7 receptors: studies in rat, mice and human. Cells. (2024):13. doi: 10.3390/cells13020161 38247852 PMC10814008

[121] Cui M Huang YL Tian CH Zhao Y Zheng JL. Foxo3a inhibits Tnf-Α- and Il-1β-induced astrocyte proliferation: implication for reactive Astrogliosis. Glia. (2011) 59:641–54. doi: 10.1002/glia.21134 21294163 PMC3747776

[122] Gajtkó A Bakk E Hegedus K Ducza L Holló K. Il-1β induced cytokine expression by spinal astrocytes can play a role in the maintenance of chronic inflammatory pain. Front Physiol. (2020):11. doi: 10.3389/fphys.2020.543331 33304271 PMC7701125

[123] Afroz S Arakaki R Iwasa T Waskitho A Oshima M Matsuka Y. Role of Cgrp in Neuroimmune interaction via Nf-Κb signaling genes in glial cells of trigeminal ganglia. Int J Mol Sci. (2020):21. doi: 10.3390/ijms21176005 32825453 PMC7503816

[124] Song YE Zhao SR Peng PY Zhang CC Liu YH Chen Y. Neuron-glia crosstalk and inflammatory mediators in migraine pathophysiology. Neuroscience. (2024) 560:381–96. doi: 10.1016/j.neuroscience.2024.10.006 39389252

[125] Oestreich LKL O'Sullivan MJ. Transdiagnostic in vivo magnetic resonance imaging markers of Neuroinflammation. Biol Psychiatry Cogn Neurosci Neuroimaging. (2022) 7:638–58. doi: 10.1016/j.bpsc.2022.01.003 35051668

[126] Beckman D Diniz GB Ott S Hobson B Chaudhari AJ Muller S. Temporal progression of tau pathology and Neuroinflammation in a Rhesus monkey model of Alzheimer's disease. Alzheimers Dement. (2024) 20:5198–219. doi: 10.1002/alz.13868 39030748 PMC11350056

[127] Morozumi T Preziosa P Meani A Albergoni M Margoni M Pagani E. Influence of cardiorespiratory fitness and MRI measures of Neuroinflammation on hippocampal volume in multiple sclerosis. J Neurol Neurosurg Psychiatry. (2024) 95:29–36. doi: 10.1136/jnnp-2023-331482 37468307

[128] Goadsby PJ Edvinsson L. The Trigeminovascular system and migraine: studies characterizing cerebrovascular and neuropeptide changes seen in humans and cats. Ann Neurol. (2004) 33:48–56. doi: 10.1002/ana.410330109 8388188

[129] Di Piero V Fiacco F Tombari D Pantano P. Tonic pain: a Spet study in Normal subjects and cluster headache patients. Pain. (1997) 70:185–91. doi: 10.1016/s0304-3959(96)03318-0 9150292

[130] Ferraro S Nigri A Demichelis G Pinardi C Chiapparini L Giani L. Understanding cluster headache using magnetic resonance imaging. Front Neurol. (2020) 11:535. doi: 10.3389/fneur.2020.00535 32695062 PMC7338680

[131] Schulte LH Haji AA May A. Phase dependent hypothalamic activation following trigeminal input in cluster headache. J Headache Pain. (2020):21. doi: 10.1186/s10194-020-01098-2 PMC7106813 32228453

[132] Christensen RH Gollion C Amin FM Moskowitz MA Hadjikhani N Ashina M. Imaging the inflammatory phenotype in migraine. J Headache Pain. (2022):23. doi: 10.1186/s10194-022-01430-y 35650524 PMC9158262

[133] Tohyama S Datko M Brusaferri L Kinder LD Schnieders JH Hyman M. Trigeminal nerve microstructure is linked with Neuroinflammation and brainstem activity in migraine. Brain. (2025) 2025:29. doi: 10.1093/brain/awaf029 PMC12233551 39873385

[134] Vollesen AL Benemei S Cortese F Labastida-Ramírez A Marchese F Pellesi L. Migraine and cluster headache – the common link. J Headache Pain. (2018):19. doi: 10.1186/s10194-018-0909-4 30242519 PMC6755613

[135] Wei DY Goadsby PJ. Cluster headache pathophysiology — insights from current and emerging treatments. Nat Rev Neurol. (2021) 17:308–24. doi: 10.1038/s41582-021-00477-w 33782592

[136] De Freitas DB Robinson CL Villar-Martinez MD Ashina S Goadsby PJ. Current and novel therapies for cluster headache: a narrative review. Pain Ther. (2024) 14:1–19. doi: 10.1007/s40122-024-00674-7 39489854 PMC11751248

[137] May A Evers S Goadsby PJ Leone M Manzoni GC Pascual J. European academy of neurology guidelines on the treatment of cluster headache. Eur J Neurol. (2023) 30:2955–79. doi: 10.1111/ene.15956 37515405

[138] Lund NLT Petersen AS Fronczek R Tfelt-Hansen J Belin AC Meisingset T. Current treatment options for cluster headache: limitations and the unmet need for better and specific treatments—a consensus article. J Headache Pain. (2023):24. doi: 10.1186/s10194-023-01660-8 37667192 PMC10476341

[139] Sen A Mukherjee A Chakravarty A. Neurological and systemic pitfalls in the diagnosis of cluster headaches: a case-based review. Curr Neurol Neurosci Rep. (2024) 24:581–92. doi: 10.1007/s11910-024-01381-8 39432226

