# Supplementary material for: Unraveling the relationship between inflammation and cluster headache
Source: Front Neurol. 2025 Apr 3;16:1548522. doi: 10.3389/fneur.2025.1548522 (PMC12003110; doi:10.3389/fneur.2025.1548522)
Supplement: Supplementary file 1 [file Image_1.pdf]

Supplementary Material

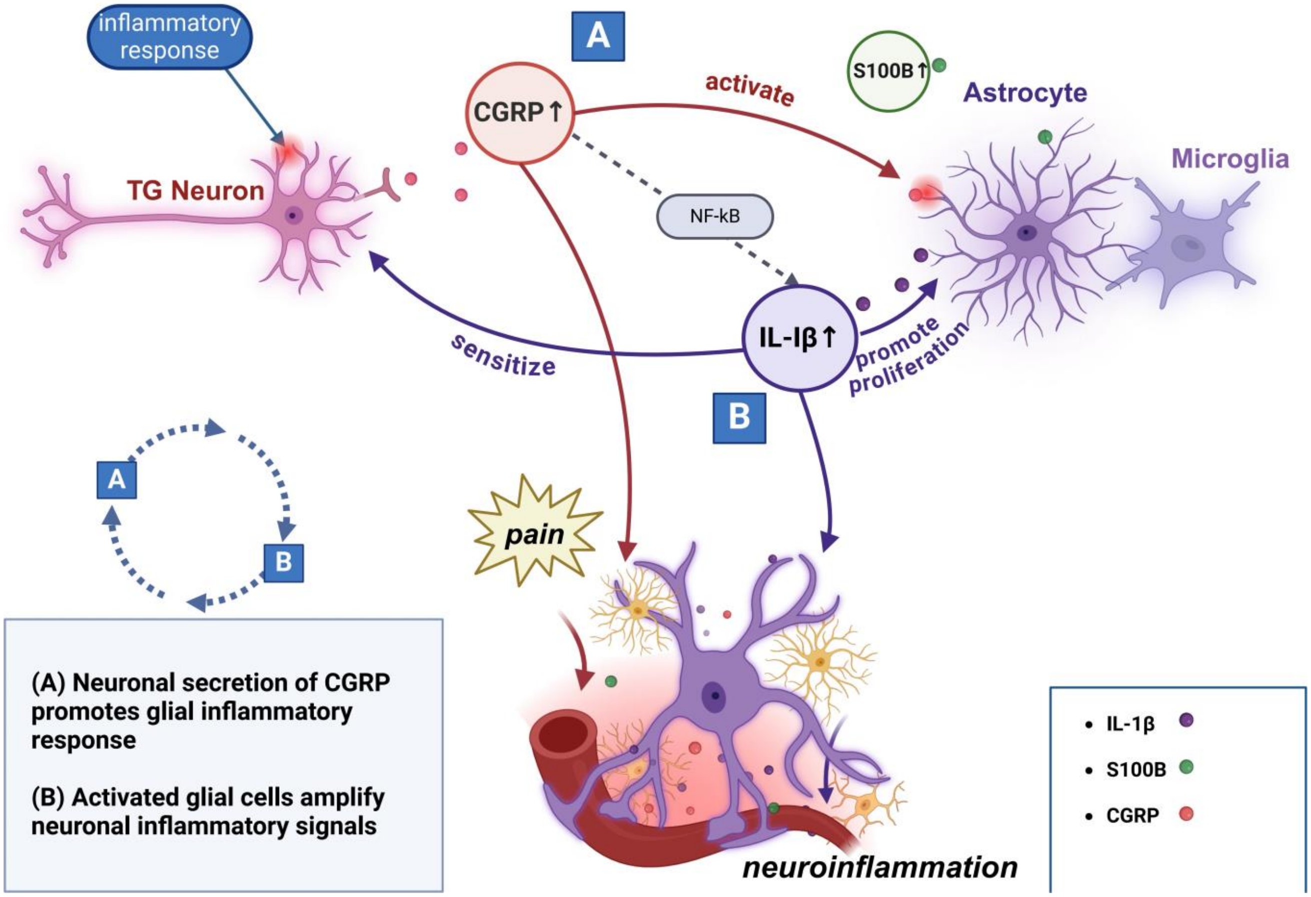

Supplementary Figure 1. Possible mechanisms of CGRP and IL-1 β in the neuron-glial inflammatory feedback loop: **(A)** Possible CGRP-mediated inflammatory initiation: Inflammatory stimuli activate trigeminal neurons, prompting CGRP release and concurrent upregulation of S100B protein expression in the target region and adjacent glial cells, which may constitute a link in the neural-glial cell interaction. Released CGRP facilitates the activation of astrocytes and microglia, subsequently inducing the secretion of inflammatory cytokines, particularly IL-1 β . Concurrently, CGRP may contribute to enhanced IL-1 β expression in glial cells by activating the NF- κ B signaling pathway; **(B)** Possible IL-1 β -mediated inflammatory amplification: As a pivotal inflammatory mediator, IL-1 β can both provide feedback to promote glial cell proliferation to intensify inflammatory responses and induce neuronal sensitization, thereby enhancing neuronal excitability and responsiveness to stimuli, increasing CGRP release and exacerbating pain perception. This process suggests this may form a feedback-regulatory relationship formed by the joint participation of CGRP and IL-1 β , characterized by progressive amplification of inflammatory signals between neurons and glial cells, thereby facilitating the persistence and expansion of inflammatory.
